# The prevalence of autism spectrum disorder traits and diagnosis in adults and young people with personality disorders: A systematic review

**DOI:** 10.1177/00048674221114603

**Published:** 2022-08-19

**Authors:** George Gillett, Laura Leeves, Amy Patel, Andreea Prisecaru, Debbie Spain, Francesca Happé

**Affiliations:** 1 Institute of Psychiatry, Psychology & Neuroscience, King’s College London, London, UK; 2 King’s College London, London, UK; 3 Social, Genetic and Developmental Psychiatry Centre, Institute of Psychiatry, Psychology & Neuroscience, King’s College London, London, UK

**Keywords:** Autism, autism spectrum disorder, personality disorder, social cognition, prevalence

## Abstract

**Objectives:**

Autism spectrum disorders and personality disorders are spectrum conditions with shared clinical features. Despite similarities, previous attempts to synthesise literature on co-existing prevalence and shared traits have employed a unidirectional focus, assessing personality characteristics of individuals with an autism spectrum disorder diagnosis. Here, we assess the prevalence of autism spectrum disorder diagnosis and/or traits among persons diagnosed with a personality disorder.

**Methods:**

We systematically reviewed the English-language literature following Preferred Reporting Items for Systematic Reviews and Meta-Analyses guidelines, according to a pre-registered protocol (PROSPERO: CRD 42021264106). Peer-reviewed quantitative studies reporting the prevalence of autism spectrum disorder diagnosis or traits in persons with an established personality disorder diagnosis were included. Studies were critically appraised using the Appraisal tool for Cross-Sectional Studies.

**Results:**

Fifteen studies were identified, including 72,902 participants (median: 48, interquartile range: 30–77). Diagnoses included borderline, schizotypal and obsessive-compulsive personality disorders, and cohorts with unspecified personality disorder diagnoses. There was significant heterogeneity in diagnostic methodology and assessment tools used. We identified preliminary evidence of an increased prevalence of co-existing autism spectrum disorder diagnosis and traits among those diagnosed with a personality disorder, although significant limitations of the literature were identified.

**Conclusion:**

Our research suggests clinicians should consider conducting a careful developmental assessment when assessing service-users with possible or confirmed personality disorder. Future research directions may include larger studies featuring clinical control groups, an exploration of shared and differentiating behavioural-cognitive features of the two conditions, and investigation into potentially shared aetiological factors. Research investigating demographic factors that may contribute to potential diagnostic overshadowing would also be welcomed.

## Introduction

Autism spectrum disorder (ASD) is a group of neurodevelopmental conditions with a prevalence of 1–2% (Roman-Urrestarazu et al., 2021). ASD is characterised by difficulties in social communication and restricted and repetitive behaviours/interests, emerging in early childhood (American Psychiatric Association, 2013). The aetiology is uncertain, although heritability is estimated between 64% and 91% with possible environmental influence (Modabbernia et al., 2017; Tick et al., 2016). Conditions including attention-deficit hyperactivity disorder (ADHD), anxiety, depression, personality disorders and psychosis are prevalent in ASD, and distinguishing between these co-occurring conditions can be challenging (Lai and Baron-Cohen, 2015; Lai et al., 2019).

Similar to ASD, personality disorders (PDs) are associated with behavioural-cognitive features and infer lifelong difficulties that can impact an individual’s functioning, social integration, relationships and education (Biskin, 2015; Gask et al., 2013). The *Diagnostic and Statistical Manual of Mental Disorders* (5th ed.; DSM-5) diagnostic criteria require an ‘enduring pattern of inner experience and behaviour that deviates markedly from the expectations of the individual’s culture’, which cannot be explained by another mental health condition (American Psychiatric Association, 2013). DSM-5 categorises PDs into three clusters: cluster A includes paranoid, schizoid, schizotypal PDs; cluster B includes antisocial (dissocial), borderline (emotionally unstable), histrionic, narcissistic PDs; and cluster C includes avoidant (anxious), dependent and obsessive-compulsive (anankastic) PDs. PDs can lead to severe distress, have high rates of comorbidity and are often highly stigmatised (Simonsen et al., 2019). The aetiology is uncertain, although both genetics and environmental factors are implicated, with heritability estimates between 30% and 80% (Fontaine and Viding, 2008).

Both ASD and PDs can be defined as egosyntonic ‘spectrum conditions’ with the spectrum representing a range of difficulties that are enduring and may be in harmony with one’s self-image. The conditions also share clinical features such as difficulties with social interactions, emotional dysregulation, impulsivity, alexithymia and self-injurious behaviour, alongside differences in cognitive style and idiosyncrasy (Lai and Baron-Cohen, 2015). Indeed, early attempts to delineate ASD used personality disorder terminology: Grunya Sukhareva described it as ‘schizoid psychopathy’, while Hans Asperger termed it ‘autistic psychopathy’, equivalent to the modern term ‘personality disorder’ (Sher and Gibson, 2021). Clinically, it can be challenging to differentiate between ASD and PD, especially in cases where individuals present for a diagnosis of ASD in adulthood, after having lived with a PD diagnosis for many years and possibly having developed camouflaging behaviours and ingrained personality traits (Cook et al., 2021; Lai and Baron-Cohen, 2015).

Distinguishing the two conditions with accurate and timely clinical diagnosis is important. Therapeutic strategies for individuals with ASD primarily consist of psychosocial interventions and environmental adaptations (Bishop-Fitzpatrick et al., 2013), whereas management of PDs typically includes psychological therapies (Budge et al., 2013; Gask et al., 2013). Delayed diagnosis may reduce the effectiveness of therapeutic interventions in both conditions (Bozzatello et al., 2019; Chanen and Thompson, 2018; Hyman et al., 2020). Furthermore, individuals with co-existing diagnoses of ASD and PD may have additional needs which require thorough assessment and management (Dell’Osso et al., 2021).

Understanding the prevalence of ASD diagnosis and traits among PD cohorts is therefore required to inform the clinical assessment and management offered to this clinical population. However, analysis of the previous literature reveals a unidirectional focus with data synthesis having been predominantly collated from cohorts previously diagnosed with ASD, neglecting the prevalence of ASD among cohorts diagnosed with PD (Rinaldi et al., 2021; Vuijk et al., 2018). When assessed in adult ASD cohorts, PDs from clusters A and C appear most strongly associated with ASD and may have a prevalence of up to 60% (Lai and Baron-Cohen, 2015; Lugnegård et al., 2012; Vuijk et al., 2018). Other reviews have been limited by assessing personality trait measures in non-clinical populations or by focusing on single PD diagnoses (Lodi-Smith et al., 2019; May et al., 2021; Vegni et al., 2021). Despite literature suggesting that diagnostic overshadowing may contribute to the potential misdiagnosis of autistic people with PD diagnoses, or that the different diagnoses may represent subgroups or precursors of each other, these concepts have not been rigorously assessed in systematic reviews to date (Lai and Baron-Cohen, 2015). While the onset of ASD precedes that of PD, the timing of diagnosis may not follow this pattern in clinical practice. A bidirectional analysis of the relationship between the two conditions is therefore necessary to explore co-prevalence and potential diagnostic overshadowing. This review aims to assess co-existing ASD diagnoses and traits in individuals with a clinical diagnosis of PD, to inform clinical assessment, diagnosis and management, and to identify directions for future research.

## Methods

We conducted a systematic review of English-language literature relating to the prevalence of ASD diagnosis and traits among people with PD. The Preferred Reporting Items for Systematic Reviews and Meta-Analyses (PRISMA) approach was followed (Page et al., 2021). The protocol detailing review question, search strategy and inclusion/exclusion criteria was pre-registered with PROSPERO (CRD 42021264106).

### Search strategy

Medline, CINAHL, EMBASE and PsycInfo were searched from inception to 29 June 2021. Search terms related to autism including ‘autis*’, ‘ASD’, ‘ASC’, ‘Asperger*’, ‘social communication’, ‘repetitive behavio*’, ‘social cognition’ and ‘mentali*’, and all personality disorder diagnoses, including ‘personality disorder’, ‘schizoid personality’, ‘schizotypal personality’, ‘paranoid personality’, ‘antisocial personality’, ‘borderline personality’, ‘histrionic personality’, ‘narcissistic personality’, ‘anankastic personality’, ‘anxious personality’, ‘avoidant personality’ and ‘dependent personality’. The full strategy is given in Supplementary Material. Authors of conference abstracts were contacted to identify further references.

### Selection criteria

The titles and abstracts of all references were independently screened for eligibility by two authors. Full texts of eligible articles were assessed by two authors and disagreements resolved by a third author. Reasons for excluding a study were documented.

Inclusion criteria consisted of peer-reviewed empirical, quantitative studies of participants with a mean age of 12 years or over, reporting the prevalence of ASD diagnosis and traits in persons with an established PD diagnosis. Case reports, commentaries, clinical guidelines and review articles were excluded. Diagnoses of ASD and PD were limited to those in keeping with standardised diagnostic criteria (International Classification of Diseases [ICD] or *Diagnostic and Statistical Manual of Mental Disorders* [DSM]), and ASD traits conceptualised as those ascertained by a previously validated scale using clinical assessment or self or proxy questionnaires. Studies reporting personality trait measures in the general population were excluded. ASD diagnoses were inclusive of ASD, Asperger’s syndrome and pervasive development disorder. Studies reporting ASD trait scales without a comparison group, previously established cut-off score or population estimate were excluded due to lack of interpretability.

### Data extraction, synthesis and critical appraisal

Relevant data were extracted using a standardised tool that included study aims, design, population, measures and diagnostic tools used, statistical analyses performed and relevant findings. A qualitative data synthesis was performed due to the heterogeneity of study populations, assessment measures and analyses. Statistics are reported in keeping with each primary study. The Appraisal tool for Cross-Sectional Studies (AXIS) tool was used to systematically critically appraise all included studies (Downes et al., 2016).

## Results

**Figure 1.**
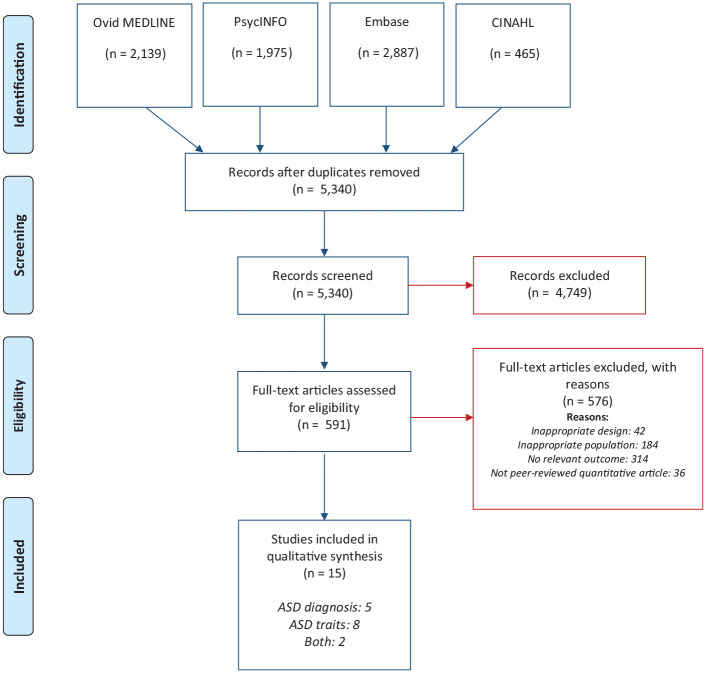
PRISMA flow diagram.

Our search returned 5340 unique references (Figure 1). The full texts of 591 articles were retrieved and 15 articles met inclusion criteria; 5 articles solely reported co-existing ASD diagnosis, 8 articles solely reported co-existing ASD traits and 2 articles reported both diagnosis and traits (Table 1).

**Table 1.** Included studies.

Author, year and country	Study aims	Study design	Sample recruitment	Population characteristics	ASD measure	Analysis	Results
*ASD diagnosis*							
Alexander et al. (2010) UK	To assess clinical differences between patients with a diagnosis of personality disorders and those without within an inpatient service for offenders with ID	Retrospective cohort	All patients treated within a 6-year period in an inpatient medium secure service for offenders with mild IDs. PD criteria: Previous clinical diagnosis of dissocial PD or EUPD. Exclusion criteria: Not stated	Overall: *N* = 138 79.0% M PD group: *N* = 77 73% M Age: 29.6 (9.4) years Dissocial PD: 68 (88.3%) EUPD: 38 (49.4%) (28 patients had both diagnoses) Non-PD group: *N* = 61 87% M Age: 31.5 (9) years	Semi-structured interview based on ICD-10 diagnostic categories (ICD-10 code F84)	Percentage of patients with co-occurring diagnosis; chi-squared testing	Fewer patients with ID and PD had co-occurring ASD diagnosis compared to patients with ID alone, 15 (19%) vs 27 (44%), *p* = 0.002
Brugha et al. (2020) UK	To validate two ASD questionnaires, AQ and RAADS-R, as screening tools in mental health settings	Cohort	Adult mental health service-users (inpatient, outpatient, and community) across 46 sites PD criteria: Clinical letters and electronic health records were reviewed to ascertain primary diagnoses of personality disorders in the 2 years prior to questionnaire completion. Exclusion criteria: Service-users in secure/elderly units, those with intellectual disability or lacking capacity to consent	Overall: *N* = 624 49.6% M Age: 42.3% < 40 years, 29.8% 40–49 years, 27.9% 50+ years PD group: *N* = 61 Demographics or specific diagnoses not described	AQ and RAADS-R. Those meeting screening criteria were assessed using ADOS	Number of patients with research-identified diagnosis of ASD who had a previous personality disorder diagnosis	Five female individuals with PD received a research-identified diagnosis of ASD, representing 8.20% of the PD sample. In comparison, three individuals with mood disorders received a diagnosis, representing 1.80%
Plana-Ripoll et al. (2019) Denmark	To assess comorbidity between mental disorders in Danish health registers, taking into account temporal relationship, age and sex	Population-based cohort	All individuals born in Denmark between January 1900 and December 2015, and living in Denmark between January 2000 and December 2016. PD criteria: Clinical diagnosis of ICD-10 personality disorder (and corresponding codes in previous ICDs) Exclusion criteria: Individuals with a developmental disorder diagnosed prior to the observation period were excluded from analyses	Overall: *N* = 5,940,778 49.8% M Age at beginning of follow-up: 32.1 (25.4) years PD group: *N* = 71,976 Demographics of subgroups not described	Clinical diagnosis of ICD F84.0 Pervasive Developmental Disorder (including ASD) in the follow-up period; information obtained the Danish Psychiatric Central Research Register (including inpatient and outpatient psychiatric facilities and emergency department records)	HR of developing a mental disorder given a prior diagnosis of another, for 90 possible pairs, using a model adjusted for age, sex and calendar time. Cumulative incidence for each pairwise association; corresponding to the percentage of individuals receiving a later diagnosis within specified time	HR of developmental disorder diagnosis following PD diagnosis: Overall: 13.7 (95% CI: [12.8, 14.7]) Male: 12.4 [11.3, 13.6] Female: 15.3 [13.9, 16.9] Cumulative incidence: At 5 years; 1.0 [0.9, 1.1] At 10 years; 1.37 [1.28, 1.47]
Rydén et al. (2008) Sweden	To assess whether ASD co-occurs with BPD in severely ill female patients, to compare clinical measures for BPD participants with and without ASD and to describe characteristic features of patients with co-existing ASD and BPD	Cross-sectional	Participants recruited from referrals to mentalisation-based treatment team (mainly from outpatient settings), patients with BPD diagnosis were invited to participate PD criteria: BPD diagnosis ascertained by structured clinical interview for DSM-IV Exclusion criteria: Male gender	Overall: *N* = 41 0% M Age: 29 (8.21) years IQ: 99.9 (11.8)	Where suspected (from general interview or observation), a clinical interview was conducted considering DSM-IV diagnostic criteria for ASD, alongside additional questionnaires; ASDI, A-TAC and FTF ASD diagnoses included autistic disorder, Asperger’s syndrome and pervasive developmental disorder NOS	Number of participants meeting diagnostic criteria reported	19 patients had possible autistic traits at general assessment and were assessed further. 6 participants (14.6% of total sample) met diagnostic criteria for ASD; 2 for Asperger’s syndrome, 4 for pervasive developmental disorder. Information was lacking for 9 patients
Shen et al. (2018) Taiwan	To develop a risk stratification model for the early diagnosis of BPD based on co-exiting psychiatric conditions	Retrospective, case control	All patients who were newly diagnosed with BPD between January 2003 and December 2006 in the Taiwanese Longitudinal Health Insurance Database (data from mandatory health insurance programme). For each BPD patient, 20 age- and sex-matched control subjects without BPD were randomly selected from the database PD criteria: BPD group received clinical diagnosis of BPD (according to ICD-9 criteria through clinical interview) within the study period Exclusion criteria: Not stated	Overall: *N* = 6132 BPD group: *N* = 292 34.9% M Age: 25 (21–33) years Control group: *N* = 5840 34.9% M Age, mean (range): 25 (21–33) years	A range of co-existing psychiatric diagnoses were diagnosed (by clinical interview using ICD-9 criteria) within 3 years were collected, including ASD	Prevalence of ASD calculated in BPD group and controls, and odds ratio of ASD diagnosis in BPD participants (vs controls) calculated	Prevalence of ASD non-significantly higher in BPD (1 (0.3%)) vs controls (2 (0.03%); *p* = 0.066. OR = 10.0, *p* = 0.066
*ASD diagnosis and traits*							
Gadelkarim et al. (2019) UK	To explore the association between OCD, OCPD and ASD in a clinical sample of treatment-seeking adult OCD patients	Cross-sectional	Patients attending a specialised OCD outpatient clinic from March 2013 to September 2014 were asked to participate; all participants fulfilled DSM-5 criteria for OCD. PD criteria: OCPD diagnosis ascertained by semi-structured interview using Compulsive Personality Assessment Scale (based on DSM-5 diagnosis) Exclusion criteria: Not stated	Overall: *N* = 67 47.8% M Age: 44.5 (11.5) years OCD + OCPD group: *N* = 24 41.7% M Age: 45.7 (11.1) years OCD only group: *N* = 43 51.2% M Age: 44.25 (12.15) years	AQ Clinical estimate diagnosis of ASD based on clinical interview based on DSM-IV criteria and assessed by a panel of expert clinicians	Independent samples t tests compared total AQ scores between OCD+OCPD and OCD group. Cohen’s effect size calculated.Chi-squared testing performed for number of participants scoring above threshold on AQ, and receiving a clinical estimate diagnosis of ASD	13 (54.2%) OCPD participants received a clinical estimate diagnosis of ASD (9 Asperger’s syndrome, 1 Autism, 3 PDD NOS), significantly higher than without OCPD group, 13 (54.2%) vs 8 (18.6%); χ^2^(1) = 12.26, *p* < 0.001. OCPD participants scored higher on the AQ (*d* = 0.78, OCPD M = 28.04, SD 7.15; without OCPD M = 22, SD = 7.92; *p* = 0.003). 16 (66.7%) OCPD participants scored >= 26 on AQ, significantly higher than without OCPD group, χ^2^(1) = 8.3, *p* = 0.004
Langmann et al. (2017) Germany	To examine the diagnostic accuracy and utility of the revised ADOS in a routine clinical sample of adolescents and adults with suspected ASD and relevant differential diagnoses (including personality disorder)	Cross-sectional	Patients referred (by clinician or self) to child and adolescent or adult outpatient clinics for specialist assessment of suspected ASD. PD criteria: PD diagnosis ascertained by Structured Clinical Interview (DSM-IV) Exclusion criteria: Severe visual, hearing or other physical or neurological impairments	Overall: *N* = 356 79% M Age: 23 (10.3) years 238 child/adolescent participants and 118 adult participants IQ: 100 (17) PD group: *N* = 92 80% M Age: 31 (13) years IQ: 113 (14.3) Non-ASD group: *N* = 191 76% M Age: 24.9 (12.1) years IQ: 102 (18)	ADOS and ADI-R	Rates of co-existing diagnosis (according to ADI-R) reported. ADOS communication and social interaction domains, revised ADOS social affect and restricted and repetitive behaviour domains and calibrated severity score (CSS) calculated for PD group	5 (5.4%) patients with PD diagnosis received co-existing ASD diagnosis using ADI-R, 87 patients did not. Communication and social interaction domain; mean: 4.22 (4.11) for non-ASD PD group vs 3.94 (3.89) for non-ASD group overall. Social affect and restricted and repetitive behaviour domain; mean: 5.23 (4.72) for non-ASD PD group vs 4.75 (4.39) for non-ASD group overall. Calibrated severity score; mean: 2.98 (2.37) for non-ASD PD group vs 2.65 (2.18) overall. Over a quarter of personality disorder participants without ASD diagnosis reached a score for the cut-off for social affect and restricted and repetitive behaviour domains of the revised ADOS (29.9% for the social affect cut-off and 25.3% for the social affect + restrictive and repetitive behaviours cut-off), higher than ADHD, conduct and emotional disorder controls
*ASD traits*							
Abu-Akel et al. (2020) UK	To examine executive functioning (response inhibition and sustained inhibition) in individuals with ASD, STPD, both diagnoses and neurotypical populations	Cross-sectional	STPD group recruited from existing study (Edinburgh High Risk Study of schizophrenia) PD criteria: STPD diagnosis made by structured clinical interview according to DSM-IV criteria. Exclusion criteria: Overall: IQ < 70, substance dependence or history of schizophrenia, schizophreniform or bipolar disorder In neurotypical group: History of, or first-degree relative with, ASD, STPD, or a psychotic illness	Overall: *N* = 88 71.6% M Age: 37.54 (10.17) years STPD: *N* = 20 70% M Age: 37.26 (9.42) years IQ: 106.40 (10.69) ASD group: *N* = 26 76.92% M Age: 39.65 (11.89) years IQ: 114.81 (16.75) Both diagnoses: *N* = 9 66.7% M Age: 35.8(10.0) years IQ: 102.44 (23.61) Neurotypical group: *N* = 33 69.70% M Age: 36.53 (9.33) years IQ: 118.06 (9.86)	Autism severity subscale of the PANSS (PAUSS)	Group differences for PAUSS (both overall scores and subscores for difficulties in social interaction, difficulties in communication and repetitive, and stereotypic patterns of behaviour) assessed using Kruskal–Wallis test with Bonferroni correction	Group differences identified for all PAUSS analyses; STPD scored higher than neurotypical group for overall PAUSS and difficulties in social interaction and difficulties in communication sub-scores, but not repetitive, and stereotypic patterns of behaviour (*p* < 0.001)
Dell’Osso et al. (2018) Italy	To assess the relationship between autistic traits and clinical features of BPD	Cross-sectional	BPD group recruited from patients with a clinical diagnosis of BPD. Non-clinical control group also recruited PD criteria: Previous clinical diagnosis of BPD Exclusion criteria: For BPD group: age below 18 years, language or intellectual impairment, comorbid schizophrenia For control group: any history of mental disorder	Overall: *N* = 119 BPD group: *N* = 50 30% M Age: 33.8 (10.0) years Control group: *N* = 69 39.1% M Age: 31.4 (11.4) years	AQ AdAS Spectrum	Mean total and domain ASD trait scores compared between diagnostic groups using Student’s *t*-test. Logistic regression with BPD diagnosis as dependent variable and AdAS Spectrum total and domain as independent variables, adjusted for age, sex and mood symptoms	BPD had higher ASD traits measured by total AQ, BPD: 20.0 (7.3) vs control: 12.9 (5.4), *p* < 0.001, and AdAS Spectrum, BPD: 51.7 (24.6) vs control: 24.0 (15.7), *p* < 0.001. BPD had higher subdomain scores for all domains, except AQ ‘attention to details’ (*p* = 0.059). In regression analysis, AdAS spectrum predicted BPD diagnosis when adjusted for age, sex and mood symptoms (*B* = 0.49; *p* = 0.002). Significant domains included ‘adherence to routine and inflexibility’ (*B* = 0.025, *p* = 0.027) and ‘restricted interests and rumination’ (*B* = 0.304, *p* = 0.006) in a separate model adjusted for age and sex only
Dell’Osso et al. (2021) Italy	To assess the presence of autistic traits in BPD, and their relationship with suicidality	Cross-sectional	BD and BPD groups recruited from patients with a clinical diagnosis of BD I/II and BPD, respectively. Non-clinical control group recruited from same university departments PD criteria: Previous clinical diagnosis of BPD Exclusion criteria: For all groups: Age < 18 or > 65 years, comorbid schizophrenia or neurodegenerative disease, language or intellectual impairment or a current substance use disorder For control group: Any history of mental disorder	Overall: *N* = 165 BPD group: *N* = 48 31.3% M Age: 34.5 (9.7) years BD group: *N* = 58 63.8% M Age: 35.5 (11.2) years Control group: *N* = 59 45.8% M Age: 32.9 (11.7) years	AdAS Spectrum	ANOVA analyses, followed by Bonferroni post hoc tests, to compare total and domain Adult Autism Subthreshold Spectrum scores between diagnostic groups	Group differences identified for all total and domain AdAS Spectrum scores (*p* < 0.001). BPD scored higher than controls for total and all subdomain scores. BPD scored lower than BD for total and all domain scores, except for the ‘childhood/adolescence’ domain. Total AdAS Spectrum scores, BPD: 51.25 (24.0) vs BD: 69.5 (27.1) vs control: 23.6 (15.1), *p* < 0.001
Dudas et al. (2017) UK	To compare autistic traits and measures of empathy and systemising in ASD, BPD, comorbid and control participants	Cross-sectional	Participants recruited from Cambridge Autism Research Database. Participants grouped by their self-report that they had previously received a formal diagnosis of ASD, BPD, both conditions, or neither condition PD criteria: Previous clinical diagnosis of BPD self-reported by participant Exclusion criteria: Criteria not stated	Overall: *N* = 2744 37.1% M Age: 39.4 (12.3) years BPD group: *N* = 23 13% M Age: 38.8 (9.3) years ASD + BPD group: *N* = 16 43.8% M Age: 36.2 (11.6) years ASD group: *N* = 624 50.2% M Age: 39.4 (13.3) years No diagnosis group: *N* = 2081 33.4% M Age: 39.5 (12.3) years	AQ	ANOVA to compare group differences between AQ scores with post hoc Games-Howell tests. Cohen’s *d* used to calculate effect size Analyses were repeated in a random sub-sample (*n* = 89; ASD = 25, No diagnosis = 25) to deal with unbalanced sample size	Full sample: Group differences identified in total AQ, BPD: 25.65 (11.3) vs ASD: 32.32 (11.2) vs ASD + BPD: 40.19 (6.1) vs no diagnosis: 17.79 (8.2); *F*_3, 2727_ = 445.65; *p* < 0.001. Overall pattern was ASD + BPD > ASD > BPD > no diagnosis. BPD vs no diagnosis had an effect size of *d* = 1.08 (*p* = 0.014), ASD vs no diagnosis had an effect size of *d* = 1.62 (*p* < 0.001) Random sub-sample: Group differences identified in total AQ, BPD: 25.65 (11.3) vs ASD: 31.4 (14.1) vs ASD + BPD: 40.2 (6.07) vs no diagnosis: 15.4(9.3); *F*_3, 85_ = 18.52; *p* < 0.001. Overall pattern was BPD > no diagnosis, ASD + BPD > BPD, BPD = ASC. BPD vs no diagnosis had an effect size of *d* = 1.1 (*p* = 0.011), ASD vs no diagnosis had an effect size of *d* = 1.51, *p* < 0.001). BPD vs no diagnosis: Cohen’s d=1.1 (p = 0.011).
Esterberg et al. (2008) USA	To assess prevalence of autistic features in adolescents with STPD, to assess the association of symptoms with autistic features, and to assess the association between autistic features and development of psychosis	Cross-sectional and prospective cohort	Adolescent participants with STPD recruited from an existing study of psychosis risk PD criteria: Diagnoses ascertained using Structured Interview for DSM-IV Personality Disorders. ‘Other PD’ group included obsessive-compulsive, schizoid, paranoid, narcissistic, borderline, avoidant and antisocial PD Exclusion criteria: Neurological disorder, mental retardation, an Axis I disorder, substance abuse/addiction	Overall: *N* = 121 Age: 14.2 (1.8) years STPD: *N* = 35 65.7% M Age: 14.2 years Other PD: *N* = 38 52.6% M Age: not stated No PD: *N* = 48 56.3% M Age: 14.0 years	ADI-R administered to adolescent’s guardian. Scores calculated for past and current functioning in three domains; social interaction, unusual interests and behaviours, and communication	MANCOVA (adjusted for sex) with *t*-tests to assess group and paired differences in autistic features between diagnostic groups	MANCOVA revealed an overall significant effect of diagnostic group on childhood, *F*(6, 198) = 4.66, *p* < 0.001, and current, *F*(6, 220) = 5.07, *p* < 0.001, autistic features. Group differences were significant for social functioning and unusual interests and behaviour domains, but not communication. Paired comparisons showed adolescents with STPD had higher mean scores on childhood social impairment, 0.99 (0.53) vs 0.44 (0.40), *p* < 0.001, and unusual interests and behaviours, 0.51 (0.38) vs 0.29 (0.38), *p* < 0.01, compared to all other adolescents, and higher mean scores on current social impairment, 0.82 (0.59) vs 0.34 (0.36), *p* < 0.001, and current unusual interests and behaviours, 0.46 (0.32) vs 0.24 (0.33), *p* < 0.001.
Esterberg et al. (2013) USA	To assess autistic traits in STPD, 22q11DS and control participants, and to assess their relationship with prodromal symptoms of psychosis	Cross-sectional	STPD and control participants were recruited from an existing study of STPD. 22q11DS participants were recruited from a case registry of patients PD criteria: STPD diagnosis confirmed by semi-structured clinical interview based on DSM-IV criteria Exclusion criteria: Axis 1 disorder (including psychosis, autism and substance use disorders) or neurological disorder	Overall: *N* = 105 54.3% M Age: 15.5 (3.5) years STPD: *N* = 30 63.3% M Age: 14.2 (1.7) years 22q11DS: *N* = 28 39.3% M Age: 19.3 (4.1) years Control: *N* = 47 57.4% M Age: 14.0 (1.9) years	Selected items of the ADI-R (administered to parents). The following domains were included: social interaction, communication and language, and repetitive, stereotyped interests and behaviours	MANCOVA analyses assessed group differences in childhood and current autistic features for each domain, controlling for sex, age and race. Partial eta squared used to indicate effect size. Post hoc comparisons performed where relevant	Childhood autistic features: Group differences identified for childhood communication (eta-squared = 0.13; *p* < 0.01), social interaction (eta-squared = 0.24; *p* < 0.001) and repetitive interests and behaviours (eta-squared = 0.12; *p* < 0.01). In post hoc comparisons, STPD had higher childhood social interaction features compared to control (STPD: 0.9(0.7) vs control: 0.4(0.4); *p* < 0.001) and higher repetitive interests and behaviours compared to controls (STPD: 0.5 (0.3) vs control: 0.3 (0.30; *p* < 0.01). There was no significant difference in communication features between STPD and controls (STPD: 0.4 (0.6) vs control: 0.3 (0.5)). Current autistic features: Group differences identified for current communication (eta-squared = 0.18; *p* < 0.001), social interaction (eta-squared = 0.21; *p* < 0.001), repetitive interests and behaviours (eta-squared = 0.12; *p* < 0.01). In post hoc comparisons, STPD had higher current social interaction features compared to controls, STPD: 0.7 (0.5) vs control: 0.3 (0.3), *p* < 0.001, and higher repetitive interests and behaviours compared to controls, STPD: 0.4 (0.2) vs control: 0.2 (0.3); *p* < 0.01. There was no significant difference in communication features between STPD and controls (STPD: 0.1 (0.3) vs control: 0.1 (0.4)).
Kaltenegger et al. (2020) Sweden	To investigate impact of autistic traits on treatment outcome of MBT for concurrent BPD and substance use disorder	Randomised controlled feasibility study	Adult participants recruited from substance dependence treatment clinic. PD criteria: DSM-IV diagnostic criteria for both BPD and substance dependence Exclusion criteria: Co-existing ASD, psychotic, bipolar 1, cognitive impairment or psychopathy diagnosis	Overall: *N* = 46 19.6% M Age: 36.7 (9.66) years	AQ	Mean AQ score and range for entire sample reported	Mean AQ score = 18.47 (range, 7–33)
Murphy (2011) UK	To examine discriminative validity of AQ in high-secure psychiatric hospital, and to examine the relationship between AQ and with cognitive function and self-reported personality traits	Cross-sectional	Male adult patients detained in a high security psychiatric hospital with psychotic disorders PD or ASD. PD criteria: Diagnosis according to DSM-IV criteria Exclusion criteria: History of head injury or neurological conditions	Overall: *N* = 105 PD group: *N* = 24 100% M Age = 37.1 (9.9) years IQ: 96.8 (13.2)	AQ	One-way ANOVA followed by independent *t* tests to assess differences in AQ total and subscale scores between patient groups.	**Mean AQ scores for PD group:** Total = 20.5 (6.5) Social skill = 3.7 (2.2) Communication = 3.1 (1.6) Attention switching: 4.9 (1.9) Attention to detail: 4.4 (2.1) Imagination: 4.5 (2.1) In PD group, one (4.2%) patient had a total AQ score above 32 and 6 (25%) above 26. No significant differences between the PD and psychotic disorder groups in AQ. ASD group scored higher in total (*d* = 1.5, *p* < 0.001), social skill (*d* = 1.4, *p* < 0.001) and communication (*d* = 1.7, *p* < 0.001) subscales.

ASD: autism spectrum disorder; PD: personality disorder; ICD-10: International Classification of Diseases, 10th Revision; AQ: Autism Spectrum Quotient; RAADS-R: The Ritvo Autism Asperger Diagnostic Scale–Revised; ADOS: Autism Diagnostic Observation Schedule; HR: hazard ratio; CI: confidence interval; BPD: Borderline Personality Disorder; DSM-IV: *Diagnostic and Statistical Manual of Mental Disorders* (4th ed.); IQ: intelligence quotient; ASDI: Asperger Syndrome Diagnostic Interview; A-TAC: Autism-Tics-Attention-deficit hyperactivity disorder and other comorbidities; FTF: Five-to-Fifteen; OR: odds ratio; OCD: obsessive-compulsive disorder; OCPD: Obsessive-Compulsive Personality Disorder; DSM-5: *Diagnostic and Statistical Manual of Mental Disorders* (5th ed.); ADI-R: Autism Diagnostic Interview–Revised; ADHD: attention-deficit hyperactivity disorder; STPD: Schizotypal Personality Disorder; PANSS: Positive and Negative Syndrome Scale; AdAS: Adult Autism Subthreshold Spectrum; ANOVA: analysis of variance; ASC: Autism Spectrum Condition; MANCOVA: multivariate analysis of covariance; ID: Intellectual disability; EUPD: Emotionally unstable personality disorder; PDD-NOS: Pervasive Developmental Disorder - Not Otherwise Specified; PAUSS: PANSS Autism Severity Score.

### Study design

#### ASD diagnosis

Of seven articles reporting prevalence of ASD diagnosis in PD cohorts, only two studies did so as a primary aim (Gadelkarim et al., 2019; Rydén et al., 2008). The remaining studies either primarily assessed the validity of ASD screening tools or assessed ASD co-prevalence in a range of psychiatric diagnoses, including PDs. Median sample size was 356 (interquartile range [IQR]: 67–6132), median age was 29.7 years (IQR: 25–32.1) and studies ranged from an exclusively female population to 79.0% male preponderance (median: 49.6% M, IQR: 34.9–79.0%). Only two articles provided data for IQ.

Four studies retrospectively assessed prevalence of ASD diagnosis made in naturalistic clinical settings (Alexander et al., 2010; Langmann et al., 2017; Plana-Ripoll et al., 2019; Shen et al., 2018) and three studies assessed prevalence of research-identified ASD diagnosis (Brugha et al., 2020; Gadelkarim et al., 2019; Rydén et al., 2008). Diagnosis was usually determined by clinical interview, sometimes in addition to supporting tools and school reports, with the exception of one study using Autism Diagnostic Observation Schedule (ADOS) scores alone (Brugha et al., 2020). Three studies assessed percentage prevalence of ASD in PD groups against a comparison group (Alexander et al., 2010; Gadelkarim et al., 2019; Shen et al., 2018) and one study reported hazard ratios (HRs) and cumulative incidence of ASD in PD groups (Plana-Ripoll et al., 2019). The remaining three studies lacked a comparison group.

#### ASD traits

Of 10 articles reporting prevalence of ASD traits, 5 studies did so as a primary aim (Dell’Osso et al., 2021; Dudas et al., 2017; Esterberg et al., 2008, 2013; Gadelkarim et al., 2019). The remaining studies primarily assessed the performance of ASD trait scales or the association between ASD traits and clinical features such as suicidality, cognitive functioning or psychotherapy treatment outcome. Median sample size was 112 (IQR: 88–165), median age was 35.5 years (IQR: 23–37.5) and studies ranged from 80.4% female preponderance to an exclusively male population (median: 51.1% M, IQR: 37.1–71.6%). Three studies provided data for IQ (median: 106.4, range: 96.8–113).

Traits were assessed using questionnaire scales in seven studies, structured assessment tools in one study and collateral interview in two studies. Trait scores were reported by subdomain in 7 of the 10 articles. The majority of studies compared ASD trait scores against a clinical (Abu-Akel et al., 2020; Dell’Osso et al., 2021; Dudas et al., 2017; Esterberg et al., 2008, 2013; Gadelkarim et al., 2019; Murphy, 2011) and/or non-clinical control group (Abu-Akel et al., 2020; Dell’Osso et al., 2018, 2021; Dudas et al., 2017; Esterberg et al., 2008, 2013), although two articles lacked statistical comparison with any control group.

### Cluster A PDs

#### Schizotypal personality disorder

Three studies assessed the prevalence of ASD traits in people with schizotypal personality disorder (STPD; Abu-Akel et al., 2020; Esterberg et al., 2008, 2013). All three studies identified increased social interaction difficulties in STPD; one study also reported increased social communication difficulties (Abu-Akel et al., 2020) and two studies reported increased repetitive and stereotyped behaviours (Esterberg et al., 2008, 2013).

Two studies analysed ASD traits using the Autism Diagnostic Interview–Revised (ADI-R). A cohort of 35 participants with STPD had higher mean scores for childhood and current social impairment and unusual interests and behaviours compared to a mixed group of adolescents with unspecified PD or no PD diagnosis, although no group differences were identified for the communication domain (Esterberg et al., 2008). In the second study, 30 STPD participants had higher mean scores for childhood and current social impairment and unusual interests and behaviours compared to a control group of 47 adolescents, although no group differences were identified for the communication domain (Esterberg et al., 2013).

One study assessed ASD traits using the PANSS Autism Severity Score (PAUSS) of the Positive and Negative Syndrome Scale in a sample of 20 adults with STPD (Abu-Akel et al., 2020). Compared to neurotypical adults, the STPD group scored higher for overall PAUSS scores and the difficulties in social interaction and communication subdomain, but not for repetitive and stereotyped behaviours. No significant differences were identified in total or subdomain scores between the STPD and autistic participants.

### Cluster B PDs

#### Borderline personality disorder

##### ASD diagnosis

Two studies reported the prevalence of ASD diagnosis in borderline personality disorder (BPD; Rydén et al., 2008; Shen et al., 2018). In a sample of 41 female patients referred for a Mentalisation-Based Treatment programme, 19 patients were suspected of having ASD based on initial clinical interview, and 6 patients met diagnostic criteria for ASD following further assessment, representing 14.6% of the total sample (Rydén et al., 2008). However, necessary data were lacking for nine patients who were excluded from further assessment. In addition, ASD was only assessed when the condition was clinically suspected, suggesting this estimate may be an underrepresentation of the sample’s true prevalence.

A retrospective case–control study of a Taiwanese health insurance database assessed the prevalence of psychiatric comorbidity across a 3-year period prior to BPD diagnosis in 292 patients newly diagnosed with BPD and 5840 controls (Shen et al., 2018). An odds ratio for ASD diagnosis of 10 was identified, although this was not statistically significant (*p* = 0.066). ASD case numbers were low in both BPD (1; 0.3%) and control (2; 0.03%) groups, suggesting a lack of statistical power.

##### ASD traits

Four studies assessed ASD traits in BPD using the AQ (Dell’Osso et al., 2018; Dudas et al., 2017; Kaltenegger et al., 2020) and AdAS (Dell’Osso et al., 2018, 2021) tools. Using the AQ, a study of 50 BPD and 69 non-clinical control participants found increased ASD traits among BPD participants in total (20.0 vs 12.9, *p* < 0.001), social skills (3.6 vs 1.5, *p* < .001), communication (3.2 vs 1.5, *p* < .001), attention switching (4.9 vs 3.2, *p* < 0.001) and imagination (3.6 vs 2.5, *p* = .001) domains. However, attention to detail domain scores did not reach statistical significance (4.7 vs 3.9, *p* = 0.059) (Dell’Osso et al., 2018). Another study assessed group differences in total AQ score between 23 adults with BPD and 3 comparison groups: those with ASD, those with no diagnosis and those with co-existing diagnoses of ASD and BPD (Dudas et al., 2017). Adults with BPD had higher mean scores than the control group (25.65 (11.3) vs 17.79 (8.2), *d* = 1.08; *p* = 0.014). The comparison between adults with BPD and those with ASD was only marginally significant (*d* = 0.51, *p* = 0.047), and not significant when the analysis was repeated in a random subsample. Adults with co-existing ASD and BPD had higher mean scores than those with ASD alone (40.19 (6.1) vs 32.32 (11.2), *d* = 0.71, *p* = 0.001), suggesting both diagnoses may have an additive effect on ASD trait scores. The remaining study using the AQ found that 46 adults with comorbid BPD and substance dependence had a mean AQ score of 18.47, although no statistical comparison was performed (Kaltenegger et al., 2020). While this mean score may be marginally higher than reported in previous validation studies, the significance of any potential difference is uncertain (Baron-Cohen et al., 2001; Ruzich et al., 2015). Furthermore, co-existing ASD diagnosis was an exclusion criterion for this study, suggesting the mean AQ score may be underestimated.

Regarding the AdAS tool, a study of 50 BPD participants found higher mean scores in total and all subdomain scores (childhood/adolescence, verbal communication, non-verbal communication, empathy, adherence to routine/inflexibility, restricted interests/rumination, hyper/hyporeactivity to sensory input; *p* < 0.001) compared to 69 non-clinical control participants (Dell’Osso et al., 2018). In logistic regression analyses, total AdAS scores predicted BPD diagnosis (*B* = 0.49, *p* = 0.002), as did subdomain scores for adherence to routine/inflexibility (*B* = 0.025, *p* = 0.027) and restricted interests and rumination (*B* = 0.304, *p* = 0.006). In another article, 48 BPD participants had higher mean total and subdomain AdAS scores compared to non-clinical controls (Dell’Osso et al., 2021). However, mean total and subdomain AdAS scores were lower for BPD participants compared to bipolar disorder participants, with the exception of the childhood/adolescence subdomain score.

#### Dissocial and borderline personality disorder

One retrospective cohort study assessed the prevalence of ASD diagnosis in participants with either dissocial or BPD, but did not present results separately by condition (Alexander et al., 2010). In a review of clinical case files of 138 offenders with an intellectual disability, admitted to a medium secure service, frequency of co-existing ASD diagnosis was lower for patients with dissocial PD or BPD compared to patients without PD, (15 (19%) vs 27 (44%), *p* = 0.002). Given the study methodology relied on clinical records rather than research-ascertained diagnoses, it is possible that diagnostic overshadowing contributed to the results. The generalisability of these findings to clinical PD cohorts is also unknown, given that all participants had a diagnosis of intellectual disability, unlike other studies included in this review.

### Cluster C PDs

#### Obsessive-compulsive personality disorder

##### ASD diagnosis

In a cohort of 67 patients diagnosed with obsessive-compulsive disorder (OCD), research-ascertained diagnosis of ASD was higher in 24 participants with coexisting diagnoses of obsessive-compulsive personality disorder (OCPD) and OCD, compared to those diagnosed with OCD alone (13 (54.2%) vs 8 (18.6%); *p* < 0.001) (Gadelkarim et al., 2019). Of note, ASD diagnosis was ascertained through ‘clinical estimate diagnosis’. Although this process was based on DSM-5 criteria, it relied on a shorter assessment (between 30 and 45 minutes) and appeared to be made without collateral history-taking or standardised tools that would ordinarily be used in clinical diagnosis. The external validity of the study’s findings beyond the context of co-existing OCD diagnosis is unclear.

##### ASD traits

The same study also reported the prevalence of ASD traits using the AQ (Gadelkarim et al., 2019). Participants with comorbid OCD and OCPD had higher total AQ scores compared to those with OCD alone (28.04 (7.15) vs 22 (7.92); *d* = 0.78, *p* = 0.003). When a cut-off score of 26 was used, 16 (66.7%) participants with co-existing OCPD and OCD scored above this threshold, significantly more than the group with OCD alone (χ^2^(1) = 8.3, *p* = 0.004).

### Unspecified PDs

#### ASD diagnosis

Three articles assessed the prevalence of co-existing ASD diagnosis in cohorts with unspecified PD (Brugha et al., 2020; Langmann et al., 2017; Plana-Ripoll et al., 2019).

One population-based cohort study used health registries of 5,940,778 individuals to identify prevalence of coexisting mental health diagnoses based on ICD-10 coding (Plana-Ripoll et al., 2019). For the 71,976 individuals with a PD diagnosis, the hazard ratio for a later additional developmental disorder diagnosis was 13.7 (95% CI: [12.8, 14.7]) and the cumulative incidence of developmental disorder was 1% (95% CI: [0.9, 1.1]) at 5-year and 1.37% (95% CI: [1.28, 1.47]) at 10-year follow-up. Hazard ratios were higher for women compared to men (15.3 (13.9–16.9) vs 12.4 (11.3–13.6)). Among those with a prior PD diagnosis, hazard ratios for developmental disorder were greater than organic, substance use, mood, neurotic, eating disorders and intellectual disabilities, but less than schizophrenia and behavioural disorders. Developmental disorder was defined according to the ICD-10 code ‘F84: Pervasive developmental disorders’. This diagnosis corresponds to ASD, with the exception of Rett Syndrome, which has a prevalence of 1 in 20,000–40,000 and is nearly always diagnosed in early childhood, making it an unlikely explanation for these results (Fu et al., 2020; Tarquinio et al., 2015).

Two articles primarily assessing the validity of ASD screening tools also reported the prevalence of co-existing ASD diagnosis in unspecified PD subgroups (Brugha et al., 2020; Langmann et al., 2017). A cohort of 624 patients were screened with the AQ and Ritvo Autism Asperger Diagnostic Scale–Revised (RAADS-R) questionnaires; those meeting screening criteria were further assessed using the ADOS instrument to confirm diagnosis (Brugha et al., 2020). Of 61 patients with a PD diagnosis, 5 females received a research-identified diagnosis of ASD, representing 8.20% of the PD sample. In comparison, 3 of 167 (1.80%) individuals with mood disorders received an ASD diagnosis, although statistical comparison was not conducted. In another study, 356 patients referred to outpatient clinics for specialist assessment of suspected ASD were assessed using the ADI-R tool (Langmann et al., 2017). Of 92 service-users with a PD diagnosis, 5 received a co-existing ASD diagnosis, representing a prevalence of 5.4%. However, the external validity of this study is unclear, given all participants had been referred following clinical suspicion of ASD.

#### ASD traits

Two studies assessed the prevalence of ASD traits in cohorts with unspecified PD (Langmann et al., 2017; Murphy, 2011). In the abovementioned study of patients referred for specialist assessment of suspected ASD, ADOS subdomain scores were also reported (Langmann et al., 2017). Over a quarter of 87 participants with PD scored above cut-off for the social affect (SA) and restricted and repetitive behaviour (RRB) domains of the revised ADOS, higher than ADHD, conduct and emotional disorder controls. However, group mean scores for the ‘communication and social interaction’ and ‘social affect and restricted and repetitive behaviour’ domains of the ADOS did not appear to differ between a group of 87 participants with PD and a larger cohort of 191 patients without ASD, although the latter group included participants in the PD subgroup and formal statistical comparison was not conducted. Furthermore, five participants with PD were excluded due to meeting diagnostic criteria for ASD, potentially limiting the validity of the mean scores.

In a separate group of 24 male patients detained in a high-security psychiatric hospital and diagnosed with a PD, mean AQ score was 20.5 (6.5) (Murphy, 2011). When cut-off scores of 26 and 32 were used, six (25%) and one (4.2%) participants scored above threshold, respectively. Compared to a group of 69 patients with psychotic disorders, no significant differences were identified. Compared to a group of 12 autistic patients, the PD group had lower total (*d* = 1.5, *p* < 0.001), social skill (*d* = 1.4, *p* < 0.001) and communication (*d* = 1.7, *p* < 0.001) AQ scores, although no significant differences were identified for the imagination, attention-switching and attention-to-detail subdomains. A regression analysis suggested that 45.7% of variability in AQ scores could be predicted by personality traits (as measured by the Millon Clinical Multiaxial Personality Inventory), with narcissistic and borderline profiles being positively associated, and histrionic and antisocial profiles being negatively associated, with total AQ score.

### Critical appraisal

Critical appraisal results using the AXIS tool are reported in Supplementary Material. Potential sources of bias included a lack of justification for sample size, unclear study recruitment methodology, recruitment from single or potentially unrepresentative clinical sites and a lack of attention to factors affecting non-response.

A number of studies reported prevalence of co-existing diagnosis or traits as secondary data rather than a primary outcome, with at least one study lacking statistical power (Shen et al., 2018). There was significant heterogeneity between and within study populations; potential confounding factors included forensic history, referral to a specialist clinic for ASD assessment and co-existing diagnoses of psychiatric disorder, substance dependence and intellectual disability. IQ data were only provided in a minority of studies, which is significant as ASD trait scales are not widely validated for populations with varying intellectual abilities (Baron-Cohen et al., 2001). Heterogeneity in gender representation existed between study populations, with results presented by gender in only one study (Plana-Ripoll et al., 2019). A number of study populations were recruited from existing research studies or databases and at least four studies appeared to analyse data from the same two populations, potentially limiting their external validity (Dell’Osso et al., 2018, 2021; Esterberg et al., 2008, 2013).

Many studies lacked control groups of participants with clinical diagnoses, making it difficult to ascertain if ASD diagnosis and traits are specifically associated with PDs as opposed to general psychopathology. There was heterogeneity in how ASD diagnosis was made, including both research-ascertained diagnoses and diagnoses derived from naturalistic clinical records systems, which may limit external validity and reliability, respectively. Studies assessing clinical records often had short follow-up periods potentially underestimating prevalence, given that diagnosis of adult ASD is often made after prolonged contact with mental health services (Fusar-Poli et al., 2022). Studies featured different assessment tools and inconsistently considered developmental and collateral information. For instance, one study formally assessed ASD diagnosis only when it was clinically suspected and developmental information was readily available (Rydén et al., 2008).

There was also heterogeneity across ASD trait scales used. ASD traits were often assessed using self-report tools, which may exhibit limitations given the prevalence of alexithymia and anosognosia in PD populations (Martin et al., 2021; Nicolò et al., 2011). Interestingly, one study demonstrated apparent disagreement between assessment tools (Langmann et al., 2017). Despite a number of participants scoring above threshold scores for current ASD traits assessed by the ADOS instrument, these were considered ‘false positive’ scores due to the ADI-R (which assesses developmental symptoms) being used to confirm diagnosis in the study. Retrospective assessment using tools such as the ADI-R may feature recall bias and less reliably detect ASD in adult populations (Fusar-Poli et al., 2017; Kamp-Becker et al., 2021). Finally, there was inconsistent reporting of subdomain trait scores, which may be important given the fractionable-triad model of autism (Happé and Ronald, 2008).

## Discussion

Our review identifies preliminary evidence of an increased prevalence of ASD diagnosis and traits among individuals diagnosed with BPD, STPD, OCPD and unspecified PD diagnoses. Regarding ASD diagnosis, all studies identified higher prevalence among PD cohorts compared to either within-study comparison groups or, where lacking, general population estimates of ASD (Bougeard et al., 2021), with the exception of one study conducted within a forensic institution (Alexander et al., 2010). This latter study may have been confounded by the co-prevalence of intellectual disability, previous offending behaviour and a high prevalence of ASD (44%) in the control group (Alexander et al., 2010). For ASD traits, PD cohorts had higher prevalence of total and subdomain ASD trait scores compared to non-clinical control populations, and a substantial proportion of PD participants scored above cut-off thresholds. However, there was no evidence of increased ASD trait scores when compared to cohorts diagnosed with bipolar or psychotic disorders (Dell’Osso et al., 2021; Murphy, 2011). No consistent pattern among the triad of ASD traits was identified when subdomain scores for social interaction, communication and restricted repetitive behaviour were analysed.

Together, this preliminary evidence suggests that individuals diagnosed with PD are more likely to meet diagnostic criteria for ASD and score higher on ASD trait measures compared to non-clinical control populations. There are many plausible reasons for this. Both conditions may share dimensional behavioural-cognitive features leading to diagnostic uncertainty and individuals fulfilling diagnostic criteria for multiple conditions. Examples might include social-emotional detachment and eccentricity (in STPD); interpersonal difficulties, affect dysregulation, alexithymia, feelings of emptiness and identity issues (in BPD); and preoccupation with details, orderliness and inflexibility (in OCPD) (Lai and Baron-Cohen, 2015). Further characterisation of the behavioural-cognitive phenotypes and distinguishing factors between conditions may clarify these nosological uncertainties. For instance, factor analysis has begun to clarify shared and condition-specific aspects of ASD and schizotypal personality (Ford et al., 2017) and the Coventry Grid aims to distinguish ASD from attachment difficulties (implicated in BPD), although it is yet to be validated (Davidson et al., 2022). Research into the aetiology of social cognition and mentalisation difficulties may prove fruitful, given the area may represent a shared neuropsychological feature of both ASD and PDs despite stemming from different research traditions (Gur and Gur, 2016; Herpertz and Bertsch, 2014; Hessels et al., 2016). Of interest is a study that identified prevalent contemporary ASD traits among PD participants despite the apparent absence of a developmental history suggestive of ASD (Langmann et al., 2017). It is unclear whether this represents the emergence of shared symptomatology at a later point in development, or from the under-reporting of early ASD behaviours by primary caregivers of PD participants.

The increased co-prevalence of PDs and ASD may also arise from shared aetiological factors in genetic, neurobiological, environmental or psychological mechanisms, leading to genuine comorbidity between the conditions. Research into shared endophenotypes of PDs and ASD, and their aetiological associations, may therefore benefit from novel approaches such as the HiTOP and RDoC methodologies (Michelini et al., 2021).

Alternatively, having undiagnosed ASD itself may predispose to the development of PD. Individuals with ASD experience higher rates of childhood maltreatment and abuse (Dinkler et al., 2017), experience a broader range of life events as traumatic (Rumball et al., 2020), experience unique challenges with attachment relationships (McKenzie and Dallos, 2017) and may engage in long-term camouflaging behaviours (Cook et al., 2021), all of which may feasibly impact personality development. One study identified a tentative association between ASD traits and exposure to abuse among individuals with BPD, suggesting that such factors may represent a possible mechanistic pathway for the association between ASD and PD (Dell’Osso et al., 2018).

It is also plausible that this review’s findings may arise, in part, from the effect of misdiagnosis. Factors including demographics (such as gender and age of presentation), clinical heuristics (such as self-injurious behaviour being perceived as a pathognomonic feature of PD (Brüne, 2016) or ASD being wrongly stereotyped as a problem with emotional empathy (Fletcher-Watson and Bird, 2020)) and other biases may lead to a diagnosis of PD being used preferentially to ASD, despite individuals fulfilling diagnostic criteria for the latter. Interestingly, studies employing researcher-led diagnostic assessment generally reported higher rates of co-prevalence compared to studies assessing co-existing ASD diagnosis from clinical records. It is unclear to what extent this may reflect bias in clinical practice, or clinical assessment offering insights which are not typically captured by structured assessment tools used in research settings. However, the literature suggests that patients diagnosed with missed ASD in adulthood are typically known to mental health services for many years prior, often have previous diagnoses of PD, and that these PD diagnoses are commonly displaced by the later ASD diagnosis rather than viewed as co-occurring (Fusar-Poli et al., 2022; Geurts and Jansen, 2012; Kentrou et al., 2021). Notably, this appears especially prevalent among women, suggesting possible diagnostic bias (Fusar-Poli et al., 2022; Kentrou et al., 2021).

Our review has urgent clinical implications. Despite significant limitations, numerous studies suggest a general trend of increased prevalence of ASD among individuals diagnosed with PD. Clinicians should therefore be mindful to consider a careful neurodevelopmental assessment when assessing and treating individuals diagnosed with a PD, given the importance of early ASD diagnosis (Elder et al., 2017) and the known under-recognition of ASD among certain groups, such as women and older adults (Lai and Baron-Cohen, 2015; Lockwood Estrin et al., 2021). This is especially important given the different treatment approaches used for each condition, and the potential harms of offering psychotherapy to primarily treat ASD (Bishop and Swendsen, 2021). Future research may better characterise the clinical needs of individuals with co-prevalent PD and ASD diagnoses, given such individuals may be at risk of adverse health outcomes including suicide (Dell’Osso et al., 2021). Research investigating service-user experiences of being diagnosed with these conditions would also be welcomed, given that clinical experience suggests ASD diagnoses generally resonate well with service-users, in contrast to PD diagnoses which are often highly stigmatised (Leedham et al., 2020; Lester et al., 2020; Sheehan et al., 2016).

Our review identifies a number of limitations of the current literature. There was a lack of well-designed studies to primarily answer our review question; evidence was often preliminary, derived from secondary data and sometimes lacked statistical power (Shen et al., 2018). Many PD diagnoses were not investigated in any study, including a surprising absence of empirical investigation into schizoid PD, despite conceptual similarities between the conditions (Wolff, 1998). Populations featured multiple potential confounding factors, unclear recruitment methodology and studies often lacked within-study statistical comparison with clinical control groups. Further studies are required to elucidate the specific associations between PDs and ASD, as methodological factors are known to impact ASD prevalence estimates (Williams et al., 2006). Importantly, only one study presented results by gender subgroups, and analysis of diagnostic patterns by gender may be a worthy focus of future research.

### Limitations

Our review methodology also exhibits limitations. Quantitative meta-analytic data synthesis was not possible given significant heterogeneity in study design, clinical and demographic characteristics of study populations, and measurement scales used. This in part reflects the general dearth of evidence in the literature, and our review highlights significant gaps for future research. Each PD diagnosis was only assessed in a handful of studies, and significant confounding factors were present, hindering comparability.

Our focus on individuals diagnosed with a categorical PD diagnosis represents another potential limitation. The reliability of such diagnoses has been questioned, and categorical diagnosis remains controversial (Jablensky, 2002; Tyrer et al., 2007, 2015). Likewise, it is unclear how meaningful it is to study groups diagnosed with unspecified PD diagnoses, given that PDs themselves are heterogeneous groupings with differing diagnostic criteria. Nonetheless, categorical diagnosis remains the current mainstay of clinical practice and is therefore an important area of study due to clinical relevance and need, interpretability and standardisation. Future research investigating the association between ASD traits and other measures of PD, including dimensional approaches recently introduced in DSM-5, would be welcomed. Finally, our focus on the English-language literature represents a limitation, and it is possible that non-English publications of relevance were overlooked.

### Conclusion

Our review identified preliminary evidence of increased prevalence of co-existing ASD diagnosis and traits among persons with PD, although the current literature exhibits significant limitations. Future research directions may include larger studies comparing the prevalence of ASD diagnosis and traits in PD cohorts to other psychiatric cohorts, assessment of the shared and distinguishing behavioural-cognitive phenotypes of ASD and PD, and identifying potentially shared aetiological factors and mechanistic pathways linking the two conditions. Clinical implications include a recommendation for clinicians to be mindful of neurodevelopmental history when assessing service-users with a PD diagnosis, given the apparently increased prevalence of co-existing ASD and related traits within this cohort.

## Supplemental Material

sj-docx-1-anp-10.1177_00048674221114603 – Supplemental material for The prevalence of autism spectrum disorder traits and diagnosis in adults and young people with personality disorders: A systematic review Click here for additional data file. Supplemental material, sj-docx-1-anp-10.1177_00048674221114603 for The prevalence of autism spectrum disorder traits and diagnosis in adults and young people with personality disorders: A systematic review by George Gillett, Laura Leeves, Amy Patel, Andreea Prisecaru, Debbie Spain and Francesca Happé in Australian & New Zealand Journal of Psychiatry
