# Supplementary material for: The prevalence of autism spectrum disorder traits and diagnosis in adults and young people with personality disorders: A systematic review
Source: Aust N Z J Psychiatry. 2022 Aug 19;57(2):181–96. doi: 10.1177/00048674221114603 (PMC9896258; doi:10.1177/00048674221114603)
Supplement: sj-docx-1-anp-10.1177_00048674221114603 – Supplemental material for The prevalence of autism spectrum disorder traits and diagnosis in adults and young people with personality disorders: A systematic review [file sj-docx-1-anp-10.1177_00048674221114603.docx]

**Supplementary Material**

The prevalence of autism spectrum disorder traits and diagnoses in adults and young people with personality disorders: a systematic review

George Gillett^1*^, Laura Leeves^2^, Amy Patel^2^, Andreea Prisecaru^2^, Debbie Spain^3†^, Francesca Happé^3†^

^1^Institute of Psychiatry, Psychology & Neuroscience, King's College London, 16 De Crespigny Park, London, SE5 8AB, UK.

ORCID ID: 0000-0002-0270-9369

^2^King’s College London, Strand, London, WC2R 2LS, UK

^3^Social, Genetic and Developmental Psychiatry Centre, Institute of Psychiatry, Psychology & Neuroscience, King's College London, 16 De Crespigny Park, London, SE5 8AB, UK

^†^Dr Debbie Spain and Professor Francesca Happé are shared senior authors on this project

*** Correspondence:**Dr George Gillett
george.1.gillett@kcl.ac.uk

**Supplementary Material 1: Search Strategy**

|  | **Search Term** | **Additional database subject headings** | | | |
| --- | --- | --- | --- | --- | --- |
|  |  | **Ovid MEDLINE** | **PsycINFO** | **Embase** | **CINAHL** |
| 1 | Personality disorder | Personality Disorders/ | Personality Disorders/ | Personality Disorder/ | Personality Disorders/ |
| 2 | Schizoid personality* | Schizoid Personality Disorder/ | Schizoid Personality Disorder/ | Schizoidism/ |  |
| 3 | Schizotypal personality* | Schizotypal Personality Disorder/ | Schizotypal Personality Disorder/ | Schizotypal Personality Disorder/ | Schizotypal Personality Disorder/ |
| 4 | Paranoid personality* | Paranoid personality disorder/ | Paranoid personality disorder/ | Paranoid personality disorder/ |  |
| 5 | Antisocial personality* | Antisocial Personality Disorder/ | Antisocial Personality Disorder/ | Antisocial Personality Disorder/ | Antisocial Personality Disorder/ |
| 6 | ASPD |  |  |  |  |
| 7 | Dissocial personality* |  |  |  |  |
| 8 | Borderline personality* | Borderline Personality Disorder/ | Borderline Personality Disorder/ | Borderline state/ | Borderline Personality Disorder/ |
| 9 | BPD |  |  |  |  |
| 10 | Emotionally unstable personality* |  |  |  |  |
| 11 | EUPD |  |  |  |  |
| 12 | Histrionic personality* | Histrionic Personality Disorder/ | Histrionic Personality Disorder/ | Histrionic Personality Disorder/ | Histrionic Personality Disorder/ |
| 13 | Narcissistic personality* | Narcissism/ | Narcissistic Personality Disorder/ Narcissism/ | Narcissism/ | Narcissistic Personality Disorder/ |
| 14 | Obsessive compulsive personality* |  | Obsessive Compulsive Personality Disorder/ |  |  |
| 15 | Compulsive personality* | Compulsive Personality Disorder/ |  | Compulsive Personality Disorder/ | Compulsive Personality Disorder/ |
| 16 | Anankastic personality* |  |  |  |  |
| 17 | Avoidant personality* | Avoidant Personality Disorder/ | Avoidant Personality Disorder/ | Avoidant Personality Disorder/ | Avoidant Personality Disorder/ |
| 18 | Anxious personality* |  |  |  |  |
| 19 | Dependent personality* | Dependent Personality Disorder/ | Dependent Personality Disorder/ | Dependent Personality Disorder/ | Dependent Personality Disorder/ |
| 20 | (1-19, OR) |  |  |  |  |
| 21 | Autis* | Autism Spectrum Disorders/ Autistic Disorder/ | Autism Spectrum Disorders/ autistic traits/ | Autism/ | Autistic Disorder/ |
| 22 | ASD |  |  |  |  |
| 23 | ASC |  |  |  |  |
| 24 | Asperger* | Asperger Syndrome/ |  | Asperger Syndrome/ | Asperger Syndrome/ |
| 25 | PDD* |  |  | pervasive developmental disorder not otherwise specified/ | Pervasive Developmental Disorder-Not Otherwise Specified/ |
| 26 | Pervasive developmental disorder | Child Development Disorders, Pervasive/ |  |  | Child Development Disorders, Pervasive/ |
| 27 | Social cognition | Social Cognition/ | Social Cognition/ | Social Cognition/ | Social Cognition/ |
| 28 | Theory of mind | "Theory of Mind"/ | "Theory of Mind"/ | "Theory of Mind"/ | “Theory of Mind”/ |
| 29 | ToM |  |  |  |  |
| 30 | Mentali* | Mentalization/ | Mentalization/ | Mentalization/ | Mentalization/ |
| 31 | Mind blindness |  |  |  |  |
| 32 | Cognitive empath* |  |  |  |  |
| 33 | Perspective taking |  |  |  |  |
| 34 | Emotion recognition* |  | Emotion Recognition/ |  |  |
| 35 | Emotion perception* |  |  |  |  |
| 36 | Affect recognition* |  |  |  |  |
| 37 | Affect perception* |  |  |  |  |
| 38 | Social communication* | Social Communication/ | Social Communication/ |  |  |
| 39 | Restricted interests |  |  |  |  |
| 40 | Repetitive interests |  |  |  |  |
| 41 | Circumscribed interests |  |  |  |  |
| 42 | Repetitive behavio* | Stereotyped Behavior/ | Repetition Compulsion/ or Stereotyped Behavior/ |  |  |
| 43 | Restricted behavio* |  |  |  |  |
| 44 | Restricted activities |  |  |  |  |
| 45 | Repetitive activities |  |  |  |  |
| 46 | RRBI |  |  |  |  |
| 47 | Central coherence |  |  |  |  |
| 48 | cognitive flexibility |  | Cognitive Flexibility/ |  |  |
| 49 | sensory hypersensitiv* or sensory hyper-sensitiv* or sensory hyposensitiv* or sensory hypo-sensitiv* or sensory sensitiv* |  |  |  |  |
| 50 | (21-49, OR) |  |  |  |  |
| 51 | (20 & 50, AND) |  |  |  |  |
| 52 | Limit 51 to Human |  |  |  |  |
| 53 | Limit 52 to English |  |  |  |  |
| 54 | Remove duplicates from 53 |  |  |  |  |

**Supplementary Material 2: Critical appraisal results using the AXIS tool for all studies**

Code; Y = yes; N = no; DK = don’t know; NA = not applicable.

| QUESTION | Abu-Akel et al; 2020 | Alexander et al; 2010 | Brugha et al; 2020 | Dell'Osso et al., 2018 | Dell'osso et al.; 2021 | Dudas et al., 2017 | Esterberg et al; 2008 | Esterberg et al., 2012 | Gadelkarin et al; 2019 | Kaltenegger et al; 2020 | Langmann et al; 2017 | Murphy et al; 2011 | Plana-Ripoll et al; 2019 | Ryden et al; 2008 | Shen et al; 2018 |
| --- | --- | --- | --- | --- | --- | --- | --- | --- | --- | --- | --- | --- | --- | --- | --- |
| INTRODUCTION: |  | | | | | | | | | | | | | | |
| 1) Were the aims/objectives of the study clear? | Y | Y | Y | Y | Y | Y | Y | Y | Y | Y | Y | Y | Y | Y | Y |
| METHODS: |  | | | | | | | | | | | | | | |
| 2) Was the study design appropriate for the stated aim(s)? | Y | Y | Y | Y | Y | Y | Y | Y | Y | Y | N | Y | Y | Y | Y |
| 3) Was the sample size justified? | N | N | N | N | N | N | N | N | N | N | N | N | N | N | N |
| 4) Was the target/reference population clearly defined? (Is it clear who the research was about?) | Y | Y | Y | Y | Y | Y | Y | Y | Y | Y | N | Y | Y | Y | Y |
| 5) Was the sample frame taken from an appropriate population base so that it closely represented the target/reference population under investigation? | DK | N | Y | Y | Y | DK | DK | DK | N | DK | N | N | Y | N | Y |
| 6) Was the selection process likely to select subjects/participants that were representative of the target/reference population under investigation? | DK | Y | Y | DK | DK | DK | DK | DK | Y | DK | N | Y | Y | Y | Y |
| 7) Were measures undertaken to address and categorise non-responders? | N | NA | Y | NA | NA | NA | NA | NA | N | NA | NA | N | NA | N | NA |
| 8) Were the risk factor and outcome variables measured appropriate to the aims of the study? | Y | Y | Y | Y | Y | Y | Y | Y | Y | Y | Y | Y | Y | Y | Y |
| 9) Were the risk factor and outcome variables measured correctly using instruments/measurements that had been trialled, piloted or published previously? | Y | N | Y | Y | Y | Y | Y | Y | Y | Y | Y | Y | Y | Y | Y |
| 10) Is it clear what was used to determine statistical significance and/or precision estimates? (e.g. p values, CIs) | Y | N | Y | Y | Y | Y | Y | Y | Y | Y | Y | Y | Y | Y | Y |
| 11) Were the methods (including statistical methods) sufficiently described to enable them to be repeated? | Y | N | Y | Y | Y | Y | Y | Y | Y | Y | Y | Y | Y | Y | Y |
| RESULTS: |  | | | | | | | | | | | | | | |
| 12) Were the basic data adequately described? | Y | Y | Y | Y | Y | Y | Y | Y | Y | Y | Y | Y | Y | Y | Y |
| 13) Does the response rate raise concerns about non-response bias? | DK | N | Y | NA | NA | NA | NA | NA | Y | NA | N | Y | N | Y | N |
| 14) If appropriate, was information about non-responders described? | NA | NA | Y | NA | NA | NA | NA | NA | N | NA | NA | N | NA | N | NA |
| 15) Were the results internally consistent? | Y | Y | Y | Y | Y | Y | Y | Y | Y | Y | Y | Y | Y | Y | Y |
| 16) Were the results presented for all the analyses described in the methods? | Y | Y | Y | Y | Y | Y | Y | Y | Y | Y | Y | Y | Y | Y | Y |
| DISCUSSION: |  | | | | | | | | | | | | | | |
| 17) Were the authors' discussions and conclusions justified by the results? | Y | Y | Y | Y | Y | Y | Y | Y | Y | Y | Y | Y | Y | Y | Y |
| 18) Were the limitations of the study discussed? | Y | Y | Y | Y | Y | Y | Y | Y | Y | Y | Y | Y | Y | Y | Y |
| OTHER: |  | | | | | | | | | | | | | | |
| 19) Were there any funding sources or conflicts of interest that may affect the authors' interpretation of the results? | N | N | N | DK | N | N | N | N | N | N | N | N | Y | N | N |
| 20) Was ethical approval or consent of participants attained? | Y | NA | Y | Y | Y | Y | DK | Y | Y | Y | DK | DK | NA | Y | Y |
